# Supplementary material for: Exploiting ferrofluidic wetting for miniature soft machines
Source: Nat Commun. 2022 Dec 23;13:7919. doi: 10.1038/s41467-022-35646-y (PMC9789085; doi:10.1038/s41467-022-35646-y)
Supplement: Supplementary file 1 — Supplementary Information [file 41467_2022_35646_MOESM1_ESM.pdf]

Supplementary Information

Exploiting ferrofluidic wetting for miniature soft machines

Mengmeng Sun[1], Bo Hao[1], Shihao Yang[1], Xin Wang[1], Carmel Majidi[2]*, Li Zhang[13456]*

*Email: cmajidi@andrew.cmu.edu; lizhang@cuhk.edu.hk

[1]Department of Mechanical and Automation Engineering, The Chinese University of Hong Kong, Hong Kong, China
[2]Department of Mechanical Engineering, Carnegie Mellon University, Pittsburgh, PA 15213, USA
[3]Chow Yuk Ho Technology Center for Innovative Medicine, The Chinese University of Hong Kong, Hong Kong, China
[4]Multi-Scale Medical Robotics Center, Hong Kong Science Park, Shatin NT, Hong Kong SAR, China
[5]Department of Surgery, The Chinese University of Hong Kong, Hong Kong, China
[6]CUHK T Stone Robotics Institute, The Chinese University of Hong Kong, Hong Kong, China

This PDF file includes:

Figs. S1 to S35

Table S1

Captions for Videos S1 to S10

Supplementary Figures

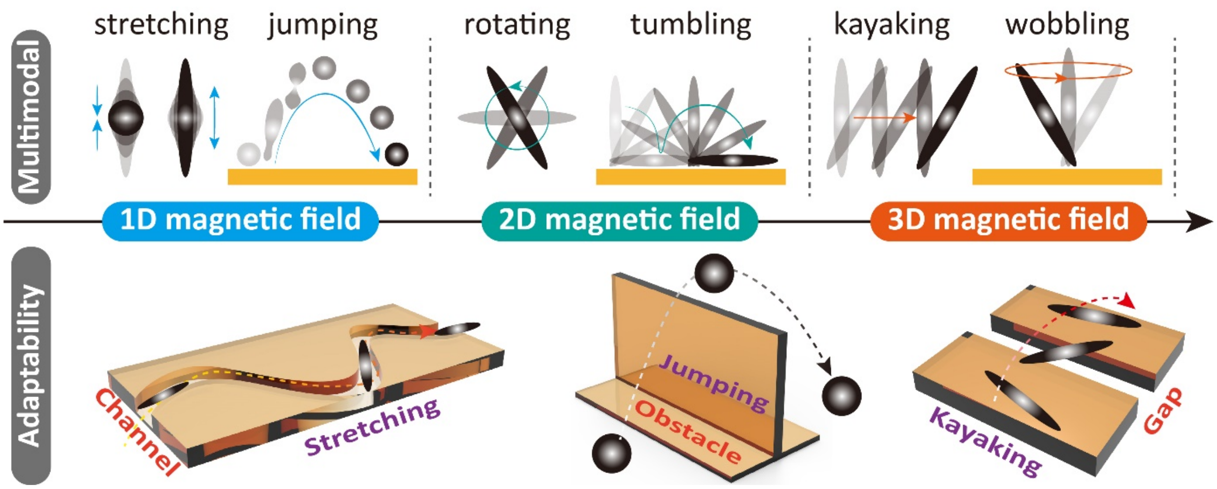

Figure S1. Multimodal locomotion and environmental adaptability of ferrofluid droplets. Schematic illustration of the six motion modes of ferrofluid droplets and their environmental adaptability.

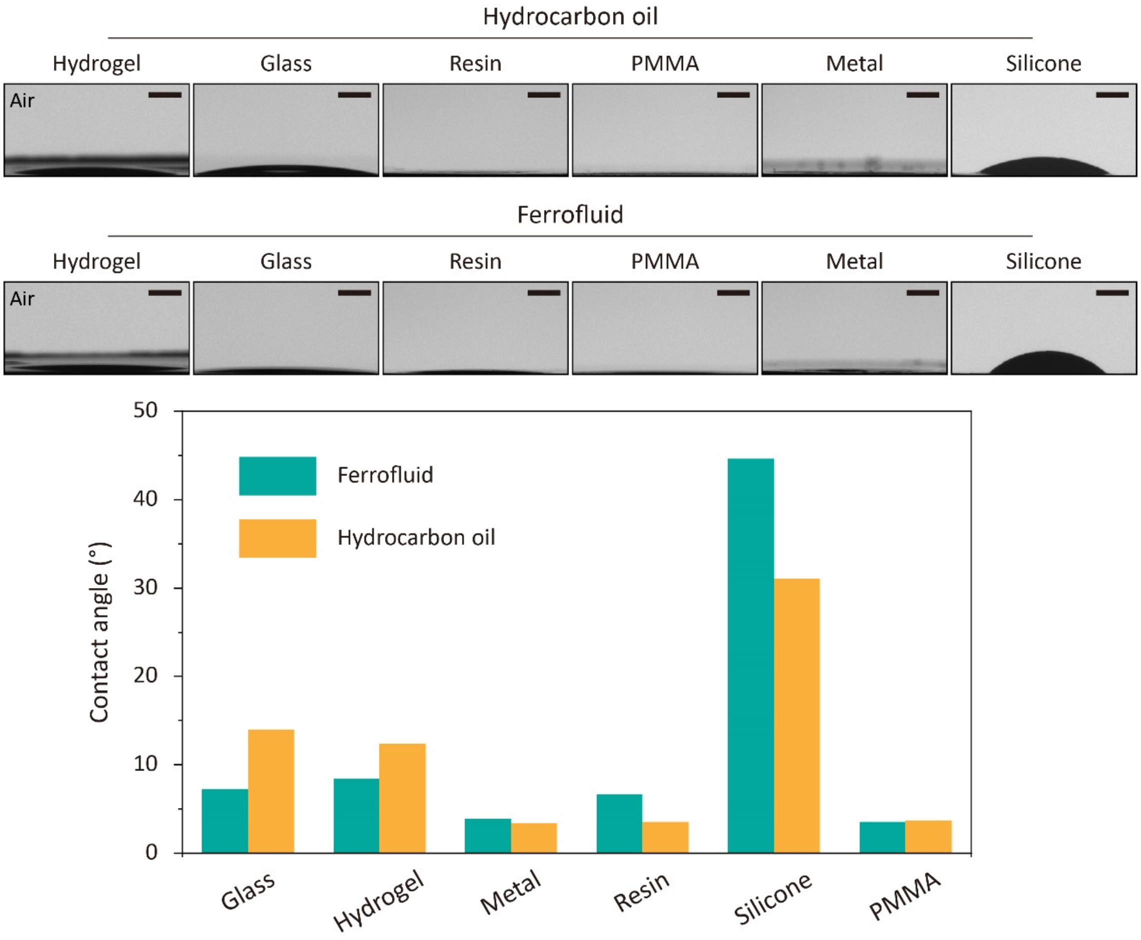

Figure S2. The wetting properties between hydrocarbon oil, ferrofluid and different surfaces in the air environment. Scale bars, 1 mm.

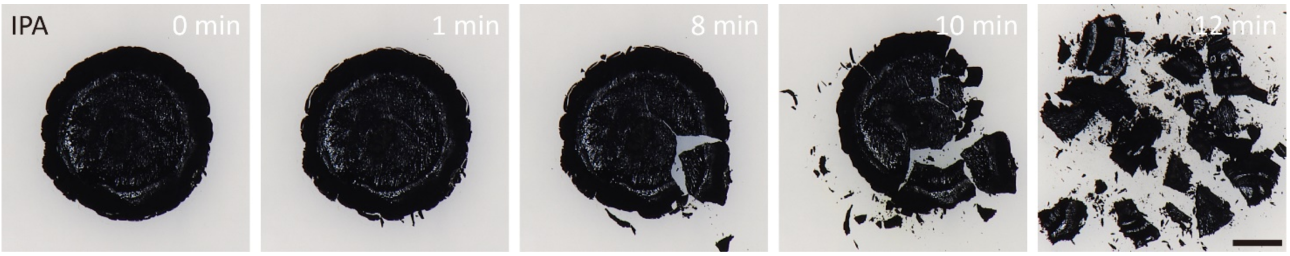

Figure S3. Photo images depicting the process of shedding and disintegration of magnetic nanoparticles deposited by the ferrofluid droplet from a silicone surface. Scale bar, 1 mm.

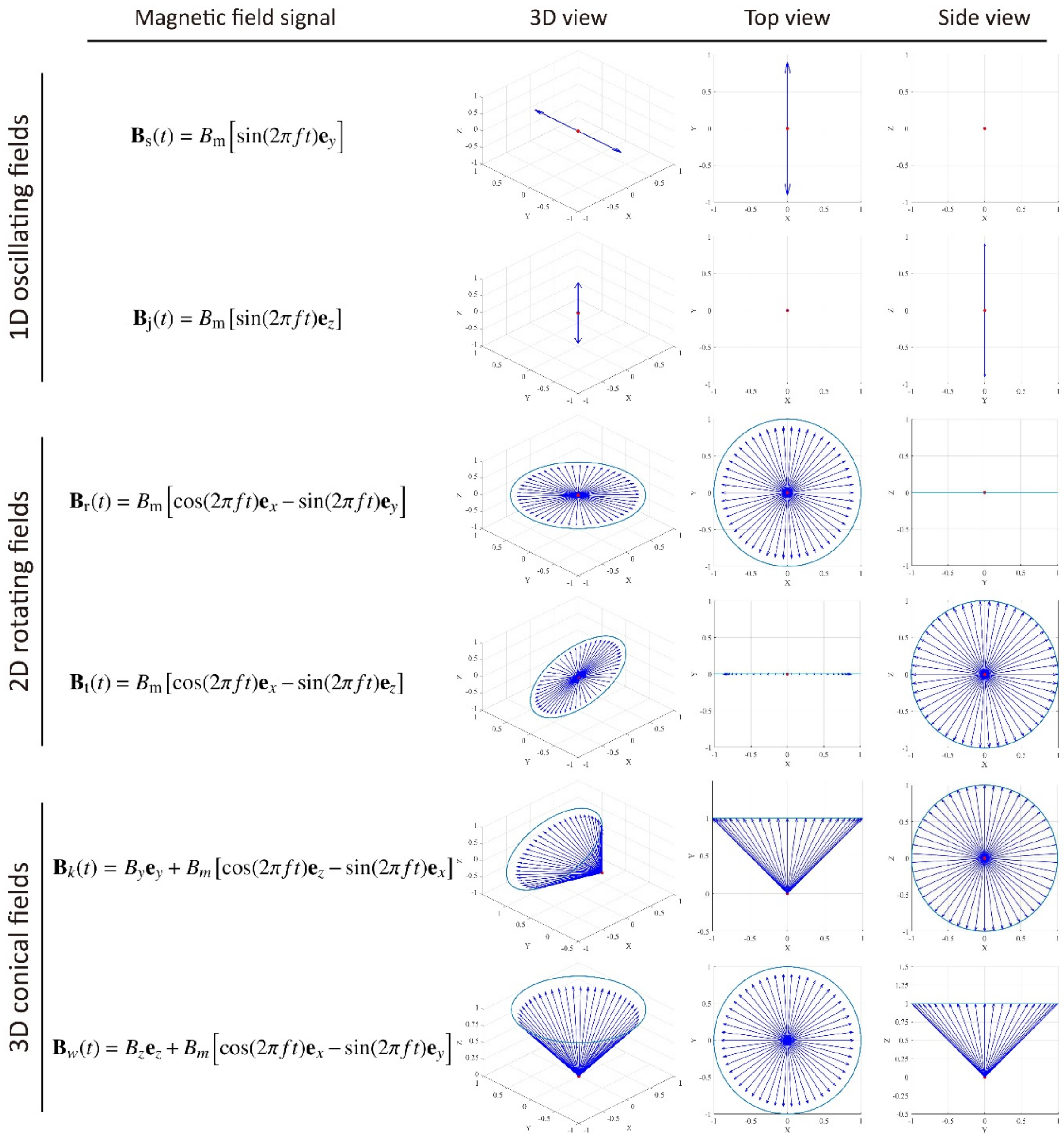

Figure S4. Visualization of the superposition of the six different magnetic fields. The superposition of the six different dynamic magnetic fields corresponding to the stretching mode ($\mathbf{B}_s(t)$), jumping mode ($\mathbf{B}_j(t)$), rotating mode ($\mathbf{B}_r(t)$), tumbling mode ($\mathbf{B}_t(t)$), kayaking mode ($\mathbf{B}_k(t)$) and wobbling mode ($\mathbf{B}_w(t)$) over a cycle, respectively. The magnetic field signal illustrates the component of the dynamic field. The 3D, top and side view illustrates the dynamic magnetic field superposition pattern.

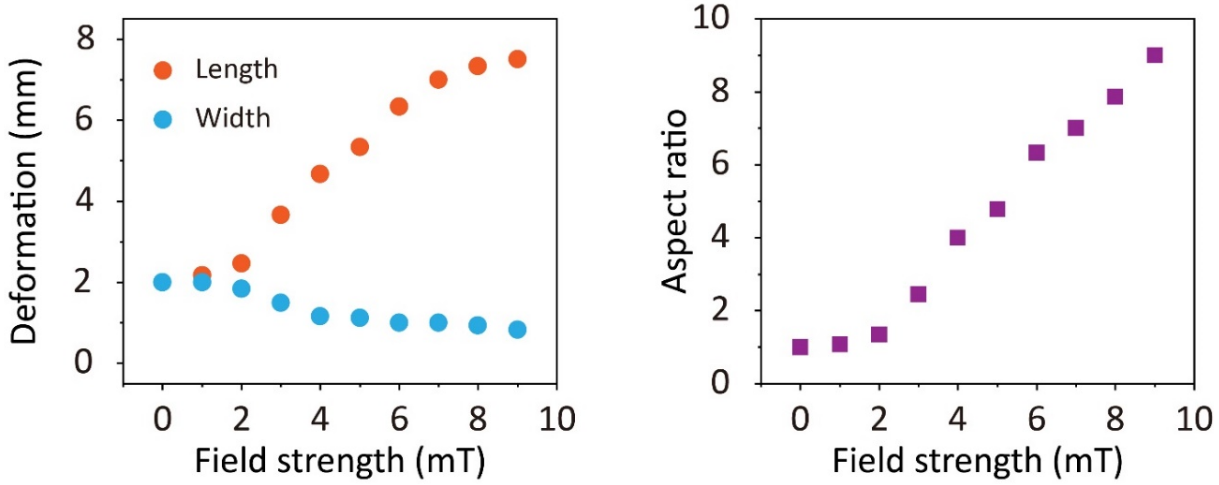

Figure S5. The aspect ratio of ferrofluid droplets in stretching mode versus magnetic field strength. The length and width of the ferrofluid droplet in the initial state are the same; both are 2 mm. The ferrofluid droplet starts to elongate with the external magnetic field strength increase, the length increases, and the width decreases. When the external magnetic field strength is 9 mT, the length of the droplet is about 7.5 mm, the width is about 0.83 mm, and the aspect ratio is about 9. Moreover, the aspect ratio of the ferrofluid droplet is proportional to the magnetic field strength.

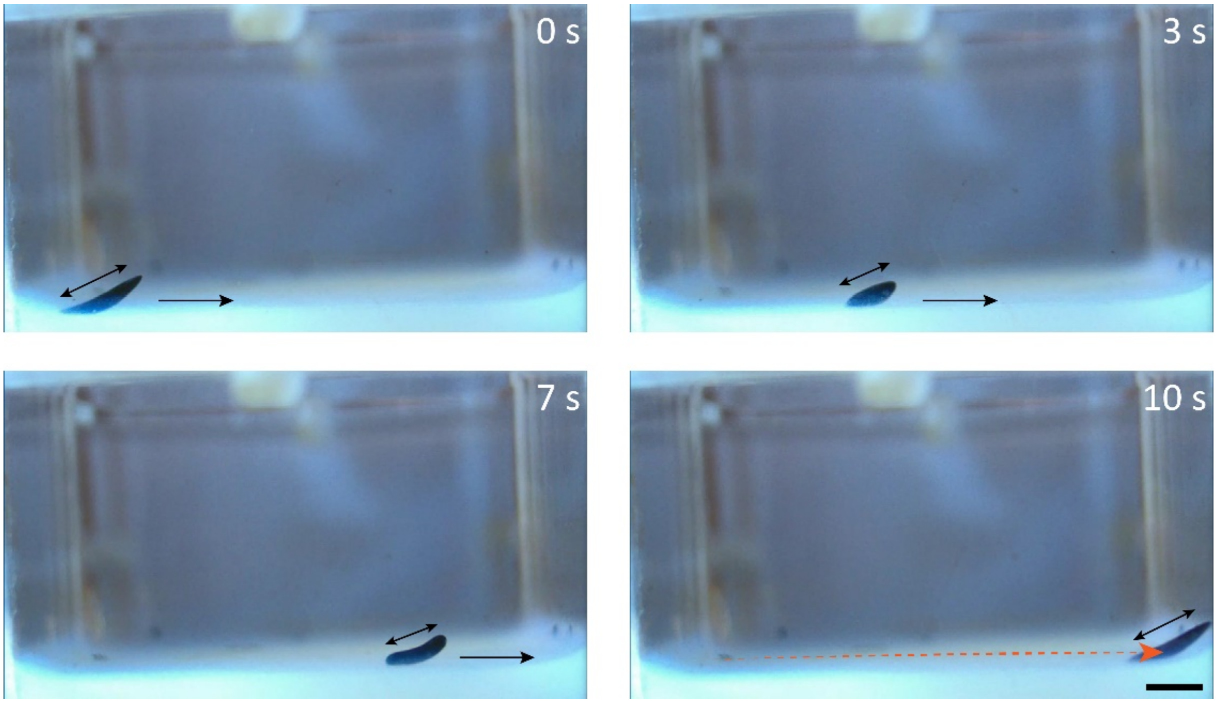

Figure S6. Translational motion of ferrofluid droplets in stretching pattern. A 1D oscillating magnetic field (frequency f = 10 Hz and magnitude Bm = 7 mT) is applied at 20°to to the substrate plane, and the ferrofluid droplet stretches back and forth in the direction of the magnetic field. The droplet translates directionally using the frictional force between the droplet and the substrate. After 10 s, the droplet moves about 55 mm. Black bi-directional arrows indicate the direction of expansion and contraction, and black single arrows indicate the direction of movement. Scale bar, 5 mm.

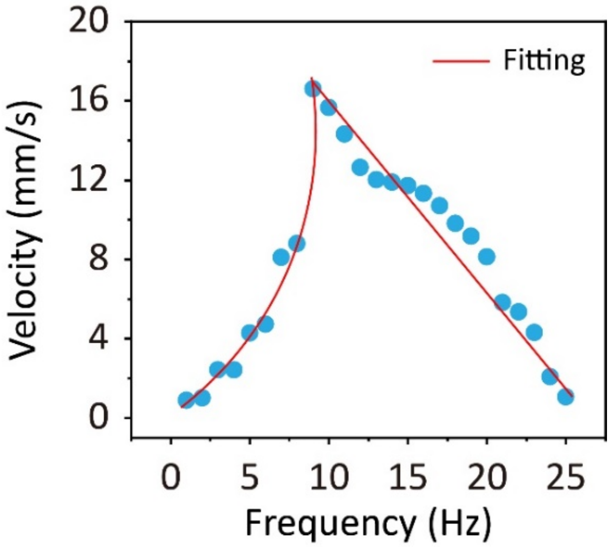

Figure S7. The velocity of the ferrofluid droplet with the stretching mode is a function of the external magnetic field. The speed of the ferrofluid droplet tends to increase and then decrease with the frequency of the external magnetic field. At a magnetic field strength of 9 mT and an angle of 30° to the substrate, the maximum translational velocity of a droplet with a diameter of 2 mm is about 16 mm/s.

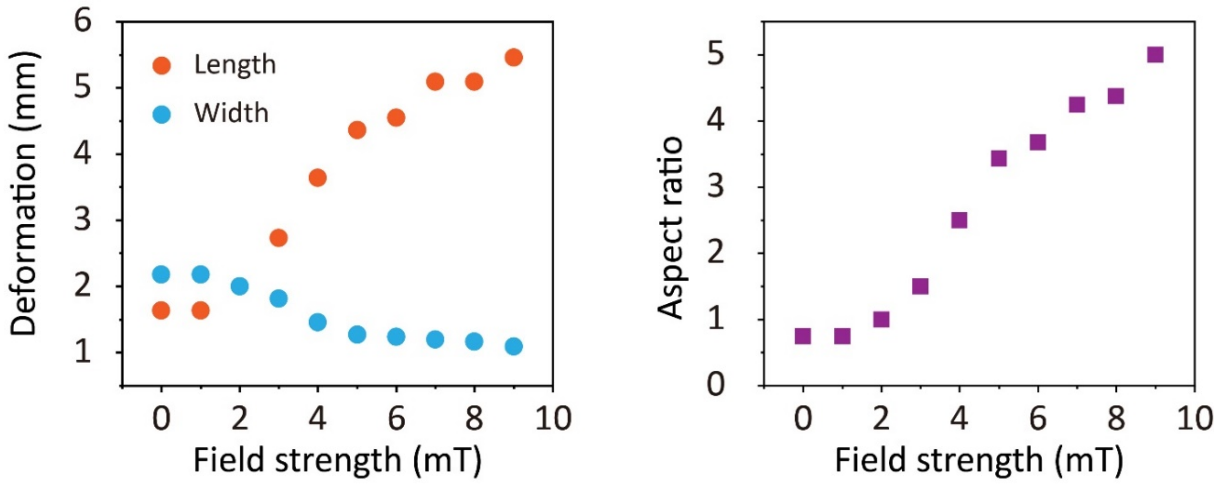

Figure S8. The aspect ratio of ferrofluid droplets in the jumping mode versus magnetic field strength. Due to gravity, the length of the droplets of ferrofluid in the initial state is slightly smaller than the width, 2 mm and 1.5 mm, respectively. With the external magnetic field strength increase, the ferrofluid droplet starts to elongate, the length increases, and the width decreases. When the outer magnetic field strength is 9 mT, the droplet length is about 5.5 mm, the width is about 1 mm, and the aspect ratio is about 5.5. Moreover, the aspect ratio of the ferrofluid droplet in jumping mode is also proportional to the magnetic field strength.

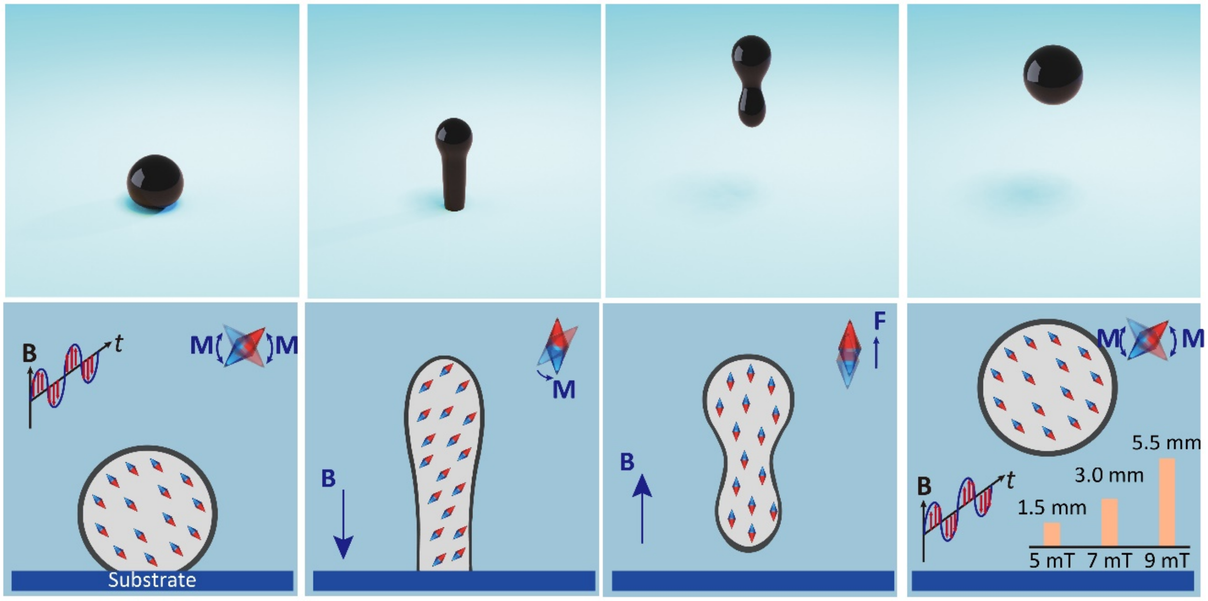

Figure S9. Mechanisms of magnetic fluid droplet jumping. The schematic diagram reveals the jumping mechanism of the ferrofluid droplet. In the initial state, the magnetic particles inside the ferrofluid droplet are magnetized and remain isotropic under the action of a high-frequency 1D oscillating magnetic field (frequency f = 100 Hz and magnitude B_m = 9 mT). And then, the opposite direction of the magnetic field is applied; thus, the repulsive force between the magnetic field and the ferrofluid droplet causes the droplet to deform significantly (due to the existence of remanent magnetization magnetic particles inside the droplet). Then the deformed ferrofluid droplet starts to contract and thus jumps up from the ground.

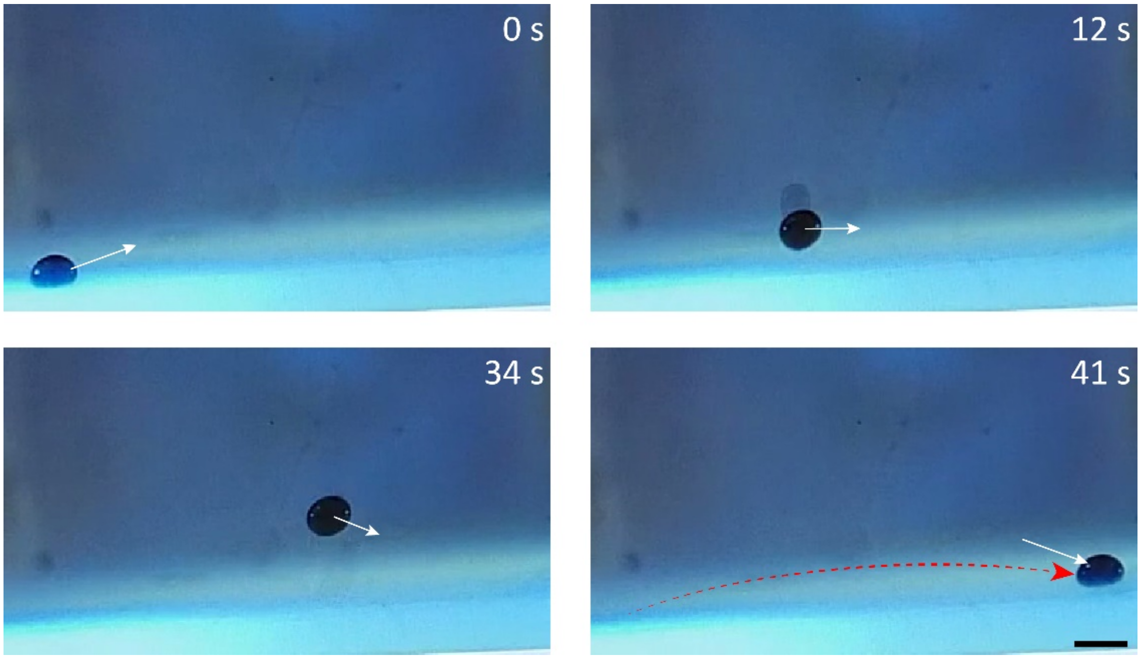

Figure S10. Snapshots of the directional jumping of ferrofluid droplet under a 1D oscillating magnetic field (frequency *f* = 100 Hz, magnitude Bm = 9 mT, angle of 80°with the substrate plane). In addition to in-situ jumping, directional jumping of ferrofluid droplets can be achieved by changing the angle between the high-frequency 1D oscillating magnetic field and the substrate plane. The ferrofluid droplet jumps from the left side to the right by 41 s. This jumping motion mode is suitable for jumping obstacles of a certain height. The white arrows indicate the direction of the ferrofluid droplet movement. Scale bar, 2 mm.

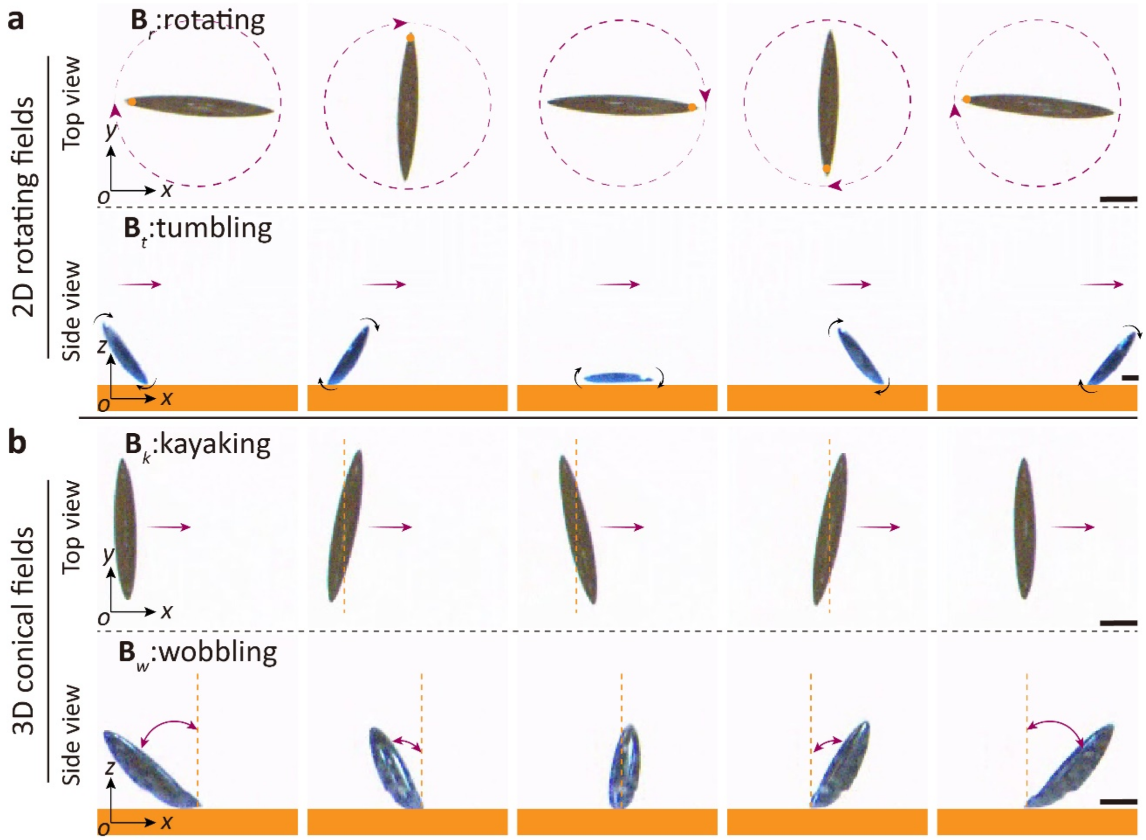

Figure S11. Locomotion modes of the ferrofluid droplet. (A) Rotating and tumbling. Ferrofluid droplets performing rotating and tumbling motion patterns under 2D rotating fields $\mathbf{B}_r(t)$ and $\mathbf{B}_t(t)$, respectively. $\mathbf{B}_r(t)$: $f = 1$ Hz and $B_m = 9$ mT. $\mathbf{B}_t(t)$: $f = 1$ Hz and $B_m = 9$ mT. **(B)** Kayaking and wobbling. Ferrofluid droplets displaying kayaking and wobbling motion modes under 3D conical fields $\mathbf{B}_k(t)$ and $\mathbf{B}_w(t)$, respectively. $\mathbf{B}_k(t)$: $f = 1$ Hz and $B_m = B_y = 9$ mT. $\mathbf{B}_w(t)$: $f = 1$ Hz and $B_m = B_z = 9$ mT. The yellow rectangle represents the substrate in the side view. When a 2D rotating magnetic field is applied, a spherical ferrofluid droplet of 2 mm diameter becomes a shuttle shape and rotates *in situ* in the *x-y* plane. The snapshots indicate that a spherical ferrofluid droplet of 2 mm diameter becomes a shuttle shape and tumbles forward in the *x-z* plane, when a 2D rotating magnetic field is applied. When the 3D conical magnetic field is applied, the fusiform ferrofluid droplet moves in the *x-y* plane in a kayaking mode, i.e., the superposition of the droplet rotating around its own axis and rotating around the *y*-axis. When a 3D conical magnetic field is applied, the shuttle-shaped ferrofluid droplet makes a circular wobbling motion around the *z*-axis. Scale bars, 2 mm.

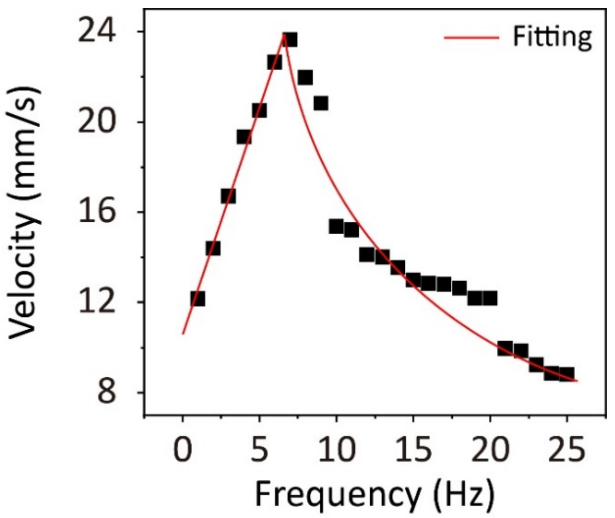

Figure S12. The velocity of the ferrofluid droplet with the tumbling mode is a function of the external magnetic field. As the frequency increases, the motion of the tumbling ferrofluid droplet changes from a synchronous state to an asynchronous state, and the translational velocity increases and then decreases, with a critical frequency of about 6 Hz. The speed of the ferrofluid droplet in tumbling mode tends to increase and then decrease with the frequency of the external magnetic field. At a magnetic field strength of 6 mT, the maximum translational velocity of a droplet with a diameter of 2 mm is about 24 mm/s. Thus this tumbling motion mode is suitable for fast long-distance maneuvers

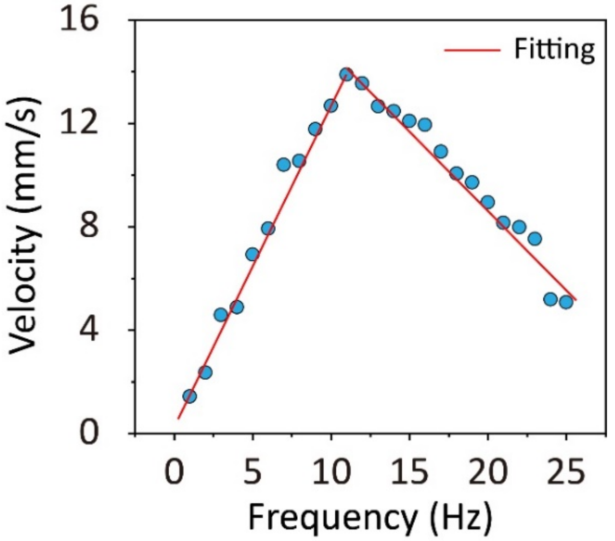

Figure S13. The velocity of the ferrofluid droplet with the kayaking mode is a function of the external magnetic field. The rate of the ferrofluid droplet in kayaking mode tends to increase and then decrease with the frequency of the external magnetic field. At a magnetic field strength of 9 mT, the maximum translational velocity of a droplet with a diameter of 2 mm is about 14 mm/s

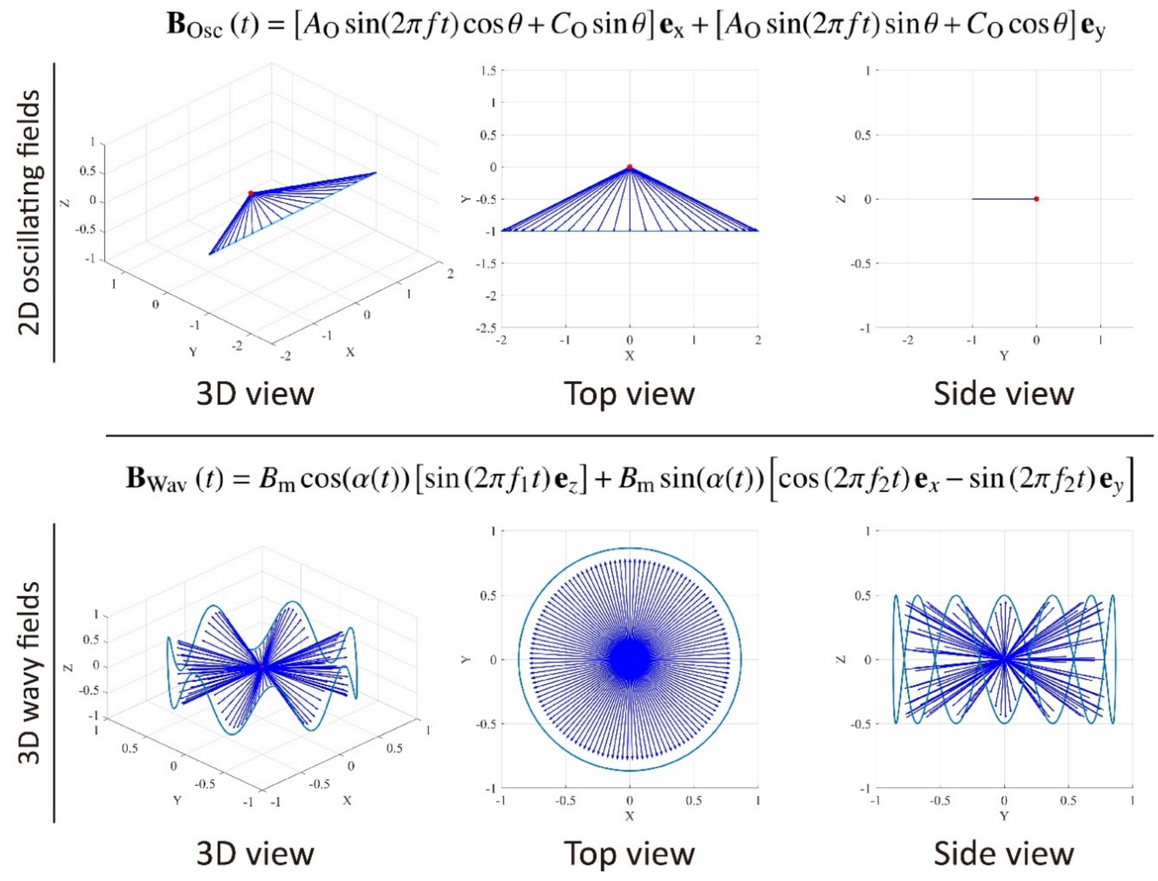

Figure S14. Visualization of the superposition of the two different magnetic fields. The superposition of the two different dynamic magnetic fields corresponds to the splitting to line and splitting to plane over a cycle, respectively. The magnetic field signal illustrates the component of the dynamic field. The 3D, top and side view shows the dynamic magnetic field superposition pattern.

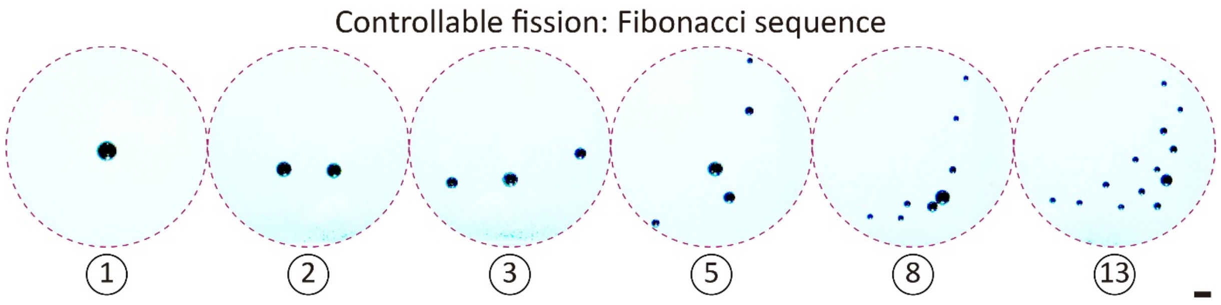

Figure S15. Controlled splitting of ferrofluid droplets. $\mathbf{B}_{wav}(t)$: $f_1 = f_2 = 30$ Hz and $B_m = 9$ mT. Scale bar, 2 mm.

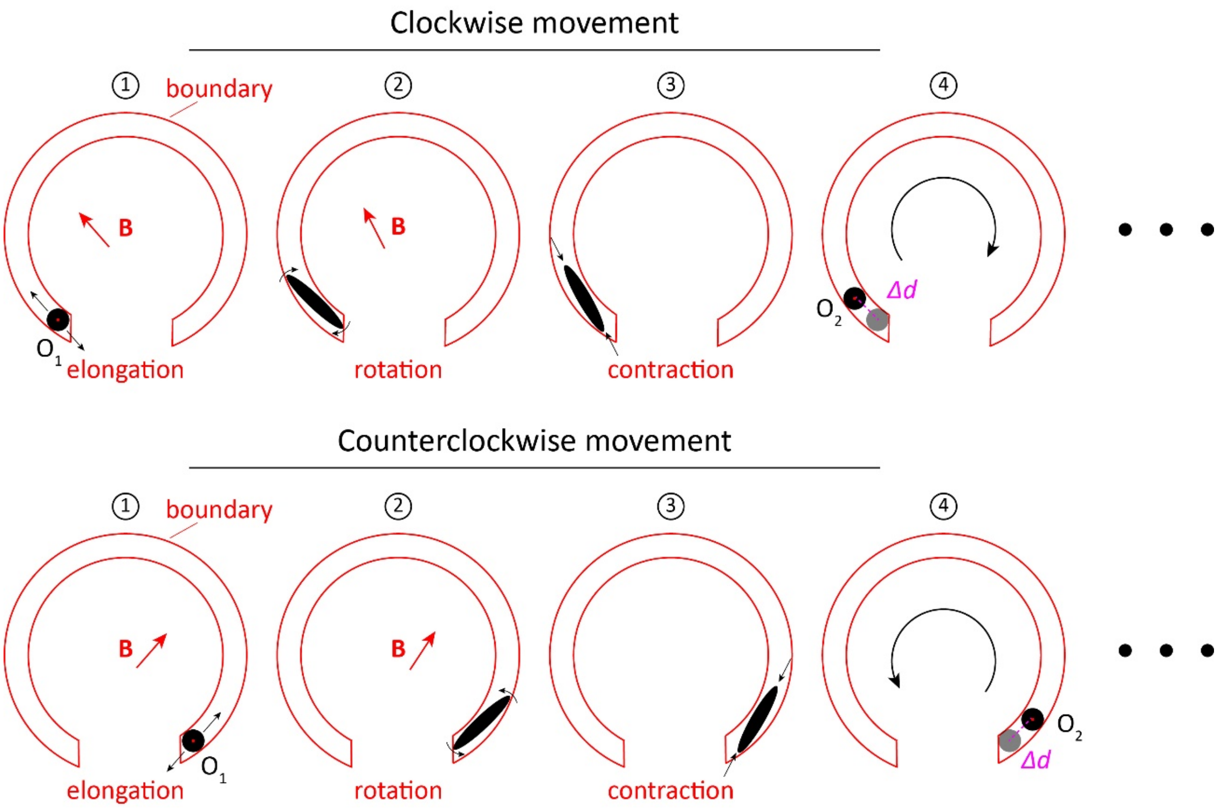

Figure S16. Mechanism for clockwise and counterclockwise movement of ferrofluid droplets in an annular channel. The black arrows indicate the direction of motion of the ferrofluid droplets and the red arrows indicate the direction of the magnetic field. When the ferrofluid droplet navigates through the annular channel and constantly stretches and shrinks, it continually changes direction with the magnetic field. The ferrofluid droplet begins to elongate under the magnetic field at the stage 1. And one end of the ferrofluid droplet will touch the side wall first and then be supported by the side wall to move forward; at stage 2, the elongated ferrofluid droplet begins to rotate within the inner walls; at stage 3, the ferrofluid droplet begins to contract. Finally, the ferrofluid droplet moves forward a small distance compared to its initial position. As the ferrofluid droplet stretches and changes direction, it follows a clockwise direction to its target position. The counterclockwise movement of the ferrofluid adopts a similar strategy.

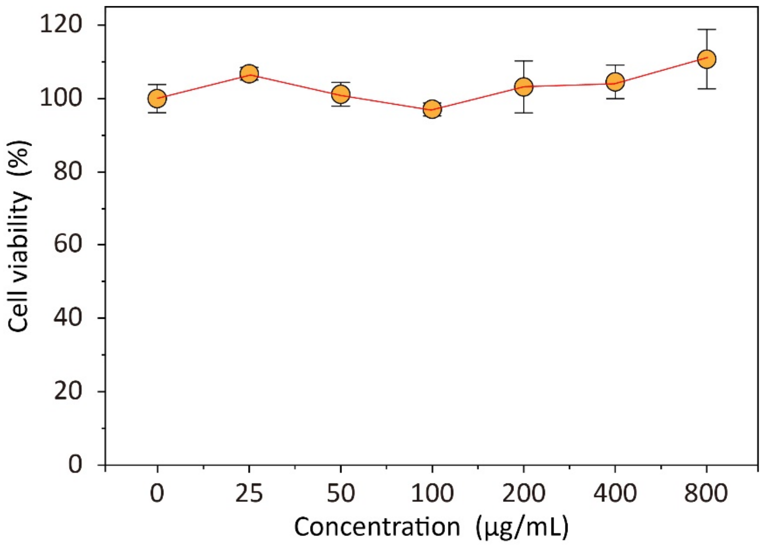

Figure S17. NIH 3T3 cells viabilities after 48 h incubation with ferrofluid suspension with different concentrations. To demonstrate the biocompatibility of our ferrofluids, we performed in vitro cytotoxicity. NIH 3T3 cells with a density of 2000 cells/well were seeded in a 96-well plate, followed by 12 h incubation in 100 µL Eagle's Minimum Essential Medium with 10% fetal bovine serum. A ferrofluid suspension was obtained by sonicating 1 mg of ferrofluid into 1 ml of fresh medium. Then the medium was discarded, and 100 µL fresh medium containing different concentrations of ferrofluid suspension was added to the NIH 3T3 cells. Subsequently, these different samples were cocultured with the cells for 48 h. The MTS assay quantified cell viability. 10 µL MTS solution was added to each well, followed by another 2 h incubation. Then nanoparticles were concentrated on the bottom with a permanent magnet, and the supernatant solution was transferred to a new 96-well plate. The absorbance was detected at 490 nm with a microplate reader. All of the tests were repeated three times. With a concentration up to 800 µg/mL, the ferrofluid was nontoxic to the NIH 3T3 cells, indicating their biocompatibility. Error bars represent standard deviation (s.d.) as n = 3.

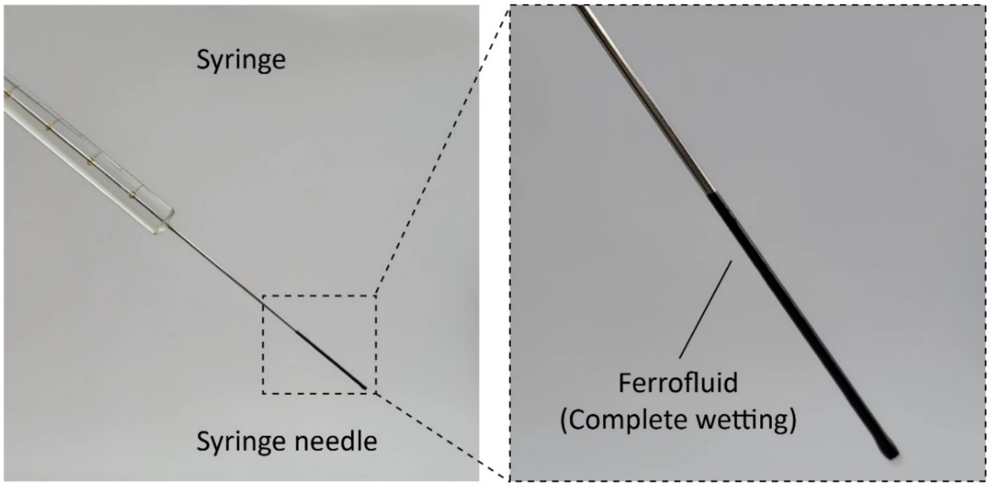

Figure S18. The syringe needle is completely infiltrated with ferrofluid. The injection of liquid cargo requires complete wetting between the syringe needle and the ferrofluid droplet. To avoid instability and ejection, a typical 5 µL droplet is injected with a liquid load of approximately 0.5 µL.

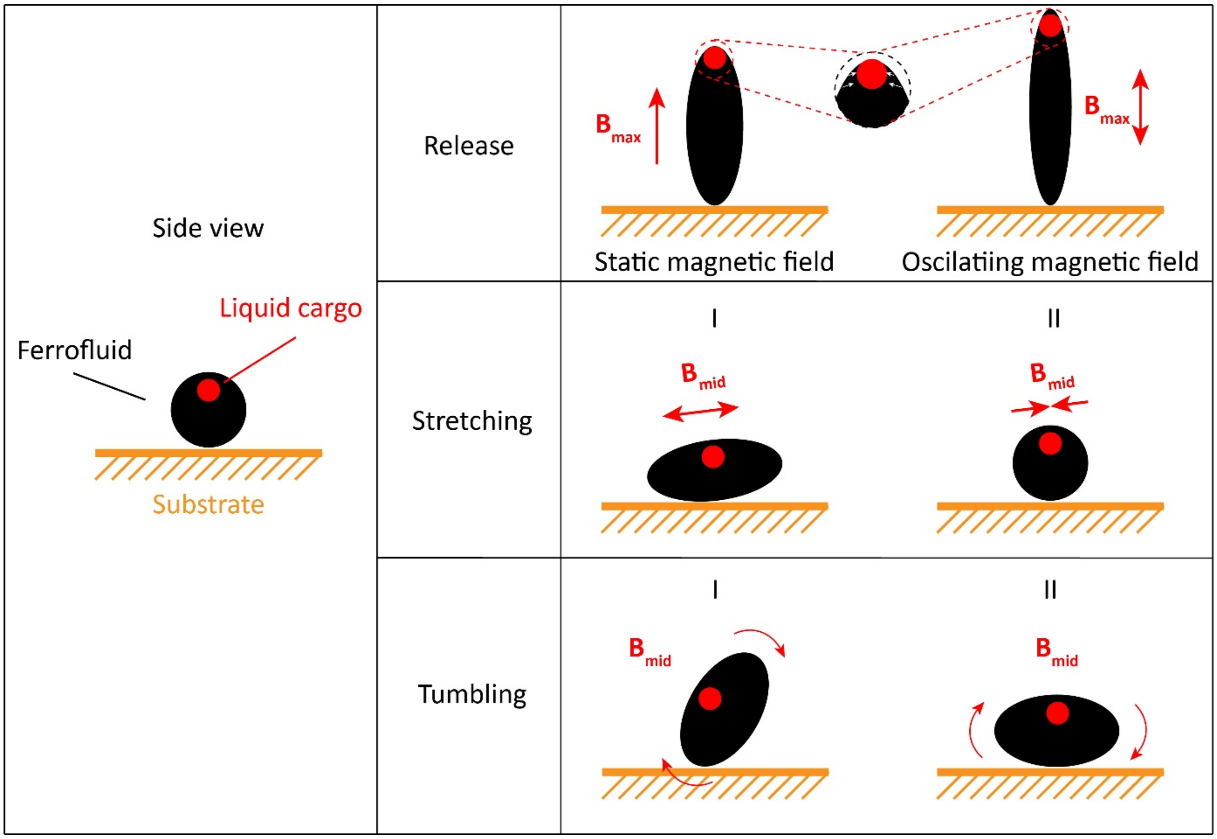

Figure S19. Mechanisms of drug release and mechanisms to maintain non-leakage during motion. The liquid capsule releases the drug by which a vertical magnetic field induces its elongation, resulting in a thinning of the ferrofluid film surrounding the liquid cargo, which then releases it. There are two critical factors in the cargo release process from liquid capsules: the first is the applied magnetic field has the maximum value of strength, and the second is that the liquid capsule is in a vertical state. The injected drug will remain above the capsule due to its density. Under the vertical magnetic field, the liquid capsule is in a standing state, and the injected cargo is located at the top of the tip of the capsule. As the strength of the vertical magnetic field continues to increase, the ferrofluid film surrounding the liquid cargo is continuously thinned. Finally, the film ruptures to release the liquid load. However, during the experiment, since our system reached the maximum magnetic field strength of 9 mT, the deformation of the ferrofluid was limited, and sometimes the liquid cargo could not be directly discharged. At this time, by applying a vertical oscillating magnetic field, the ferrofluid droplets are continuously extended and contracted. The ferrofluid droplet is significantly deformed under the action of inertial force, and then the membrane is ruptured to release the liquid cargo.

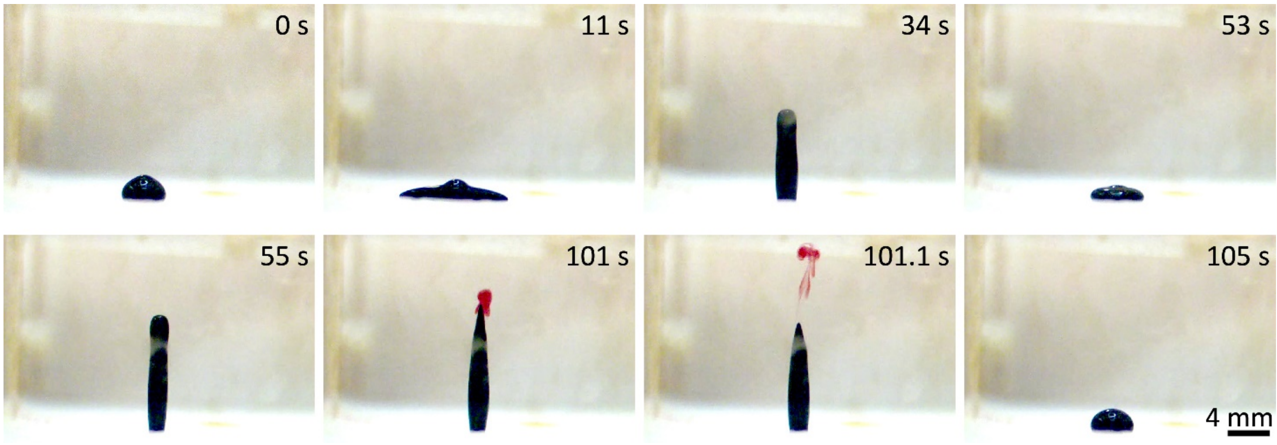

Figure S20. Liquid capsule maneuvers in various motion modes while carrying liquid and ejects on demand. First, an injector injects water-based liquid into the liquid capsule. The apparent presence in the liquid capsule is the liquid cargo (*t* = 11 s). Then, the liquid capsule maintains stability without rupture in the stretching and rotating motion pattern. Then a vertical oscillating magnetic field is applied (*f* = 1 Hz, Bm = 9 mT), which causes the liquid capsule to contract, increasing internal pressure, and the cargo is ejected from the capsule-like cuttlefish ejecting ink. Scale bar, 4 mm.

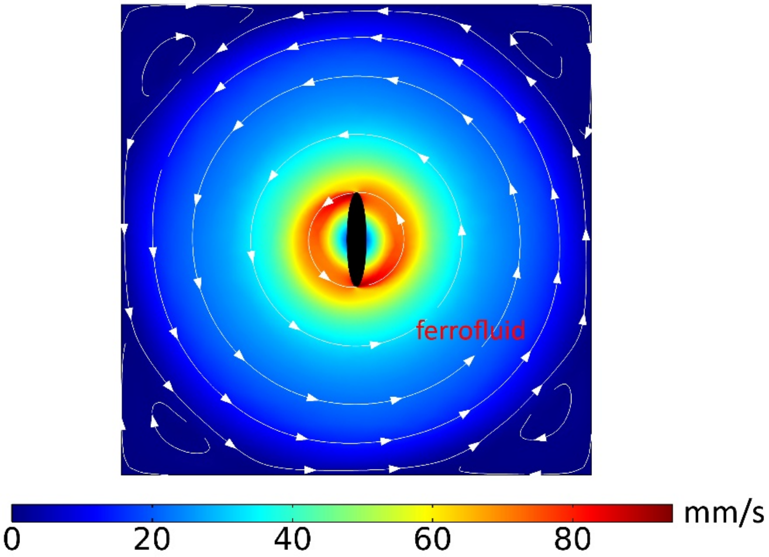

Figure S21. Fluid field distribution around the rotating liquid capsule. When a liquid capsule releases a solid cargo, it can use its rotational motion to induce a fluid field, accelerating the solid cargo's dissolution. The fluid field simulation of the rotating liquid capsule is performed using the rotating machinery module of COMSOL Multiphysics. The simulation results show that the 2 mm liquid capsule deforms into a 9 mm long shuttle shape under the action of the rotating magnetic field (*f* = 3 Hz, Bm = 9 mT). The maximum velocity of the induced generated fluid field can reach about 100 mm/s. The black area indicates the liquid capsule, and the white indicates the flow line.

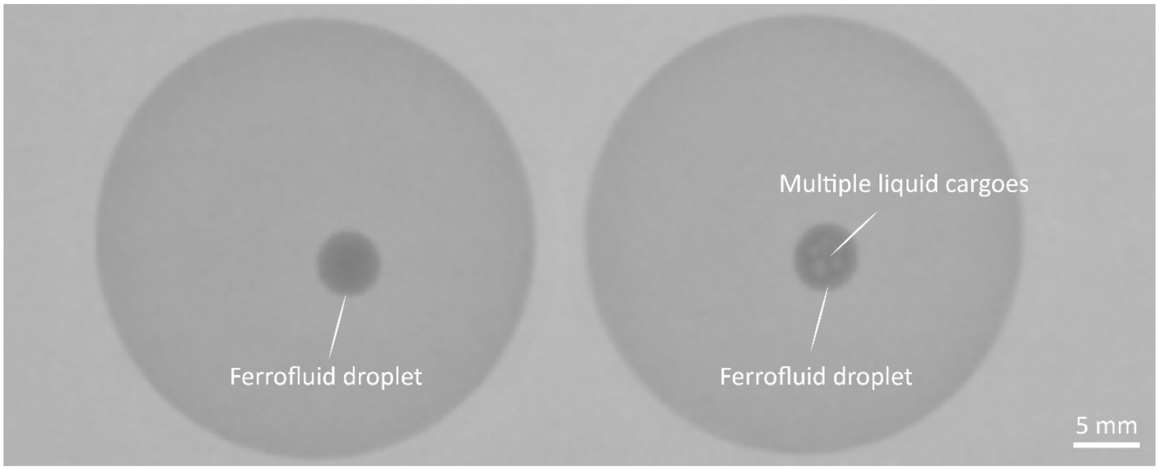

Figure S22. X-ray fluoroscopy image of a liquid capsule with multiple liquid cargoes inside. The liquid capsule allows multiple water-based cargoes to be stably held within itself. The black part represents the liquid capsule, and the white part indicates the presence of five liquid cargoes. And the surface tension between oil and water can prevent the leakage of water-based cargo. Scale bar, 5 mm.

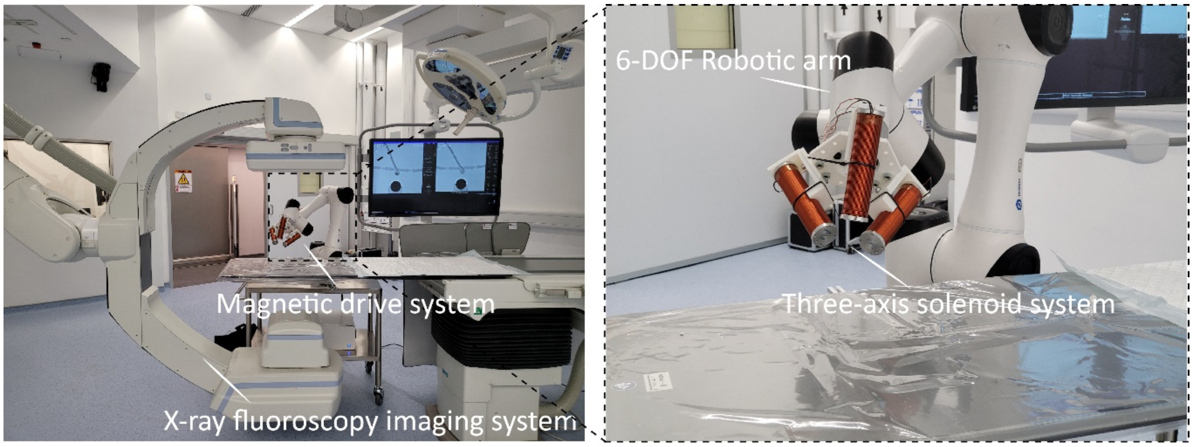

Figure S23. X-ray fluoroscopy imaging-guided magnetic drive system device. The X-ray fluoroscopy imaging-guided magnetic drive system consists of an X-ray fluoroscopy imaging system and a magnetic drive system. The X-ray imaging system is a medical clinical imaging device. The magnetic drive system consists of a 6-DOF robotic arm carrying a tri-axis electromagnetic coil. The 6-DOF robotic arm has ample working space. The three-axis electromagnetic coil system can generate a uniform magnetic field and gradient magnetic field. Realize multiple driving motions of the liquid capsule.

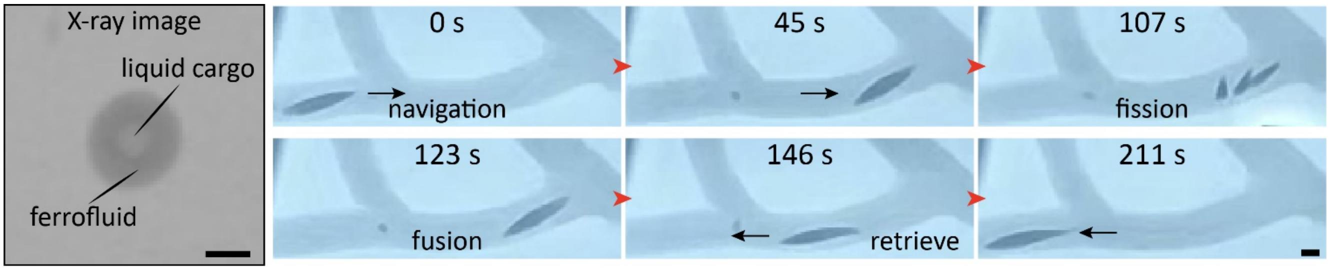

Figure S24. X-ray fluoroscopy-guided locomotion of the liquid capsule in a bile duct phantom. Under the real-time navigation of X-ray fluoroscopy imaging, the liquid capsule moves along the inner wall of the bile duct phantom driven by the gradient force of the magnetic field, and after 107 s, the capsule reaches the target position. With increasing the gradient force, the liquid capsule starts to split and release the loaded cargo. Then the magnetic field gradient is reduced, and the liquid capsule becomes the shuttle shape and then returns to the starting position. Scale bar, 2 mm.

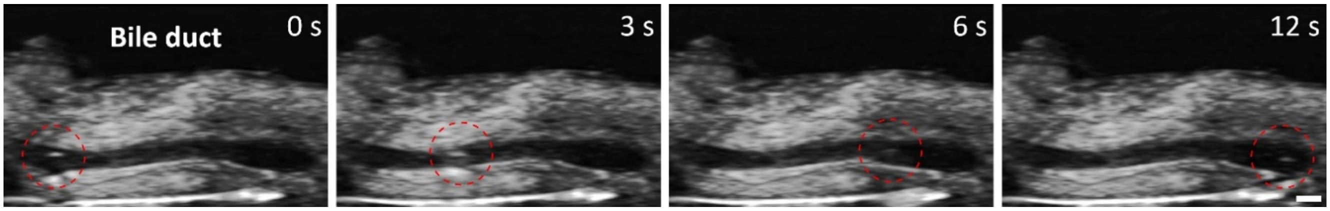

Figure S25. B-mode ultrasound image showing the navigation of the liquid capsule in the bile duct. By injecting water inside the bile duct in advance, the fluid field generated by the liquid capsule as it moves inside the bile duct can be captured by the Doppler mode of ultrasound and is used for real-time tracking and navigation. In addition, since the acoustic impedance coefficient of the liquid capsule is significantly different from that of the bile duct tissue, the B-mode of ultrasound, which depends on the acoustic impedance gradient, can also be used to track the liquid capsule and achieve its motion control and path planning based on this. At $t = 0$ s, the liquid capsule is injected inside the bile duct, forming a solid bright spot. The liquid capsule moves in a tumbling mode inside the bile duct under the action of the rotating magnetic field. Ultrasound imaging guides its motion along the inner wall of the bile duct and moves 22 mm in 12 s. Scale bar, 2 mm.

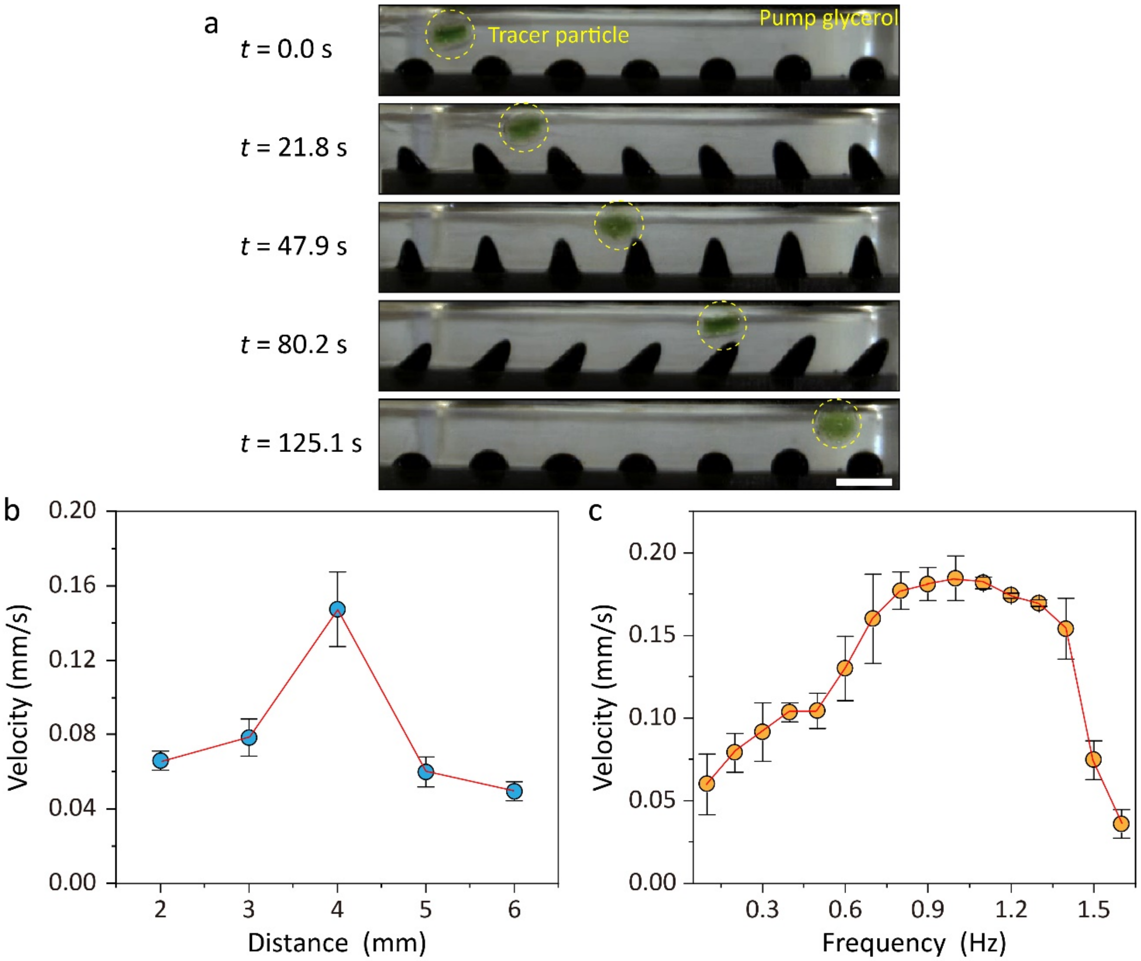

Figure S26. Liquid cilia array for pumping viscous fluids. a. Viscous fluids (glycerol) pumping by the array of 7 liquid cilia visualized by particle transportation. Scale bar: 2 mm. b, The pumping velocity versus the periodicity of the cilia array. c. The pumping velocity versus the beat frequency of the liquid array. Here, we used glycerol to create a low Reynolds number environment and investigated the relationship between the velocity of the pumped fluid and the periodicity and the beat frequency of the array. As shown in Figure S26a, we demonstrate that the array of 7 cilia can efficiently transport tracer particles in a glycerol environment (The tracer particles is hydrogel sphere with a diameter of about 2 mm, B = 9 mT, f = 0.5 Hz). When the spacing between the liquid cilia was changed from 2 mm to 6 mm (B = 9 mT, f = 0.8 Hz), the fluid velocity first increases and then decreases, and the fluid rate is the largest at 4 mm spacing, about 0.15 mm/s (Figure S26b). The relationship between the fluid velocity of the liquid cilia array and the beat frequency is shown in Figure S26c. The fluid rate gradually increases as the frequency increases and then gradually decreases. When the frequency is 1.1 Hz, the pumping velocity of the liquid ciliary array is about 0.195 mm/s (B = 9 mT, Oscilating angle = 120°). Error bars in both graphs b, c represent standard deviation (s.d.) as n = 3.

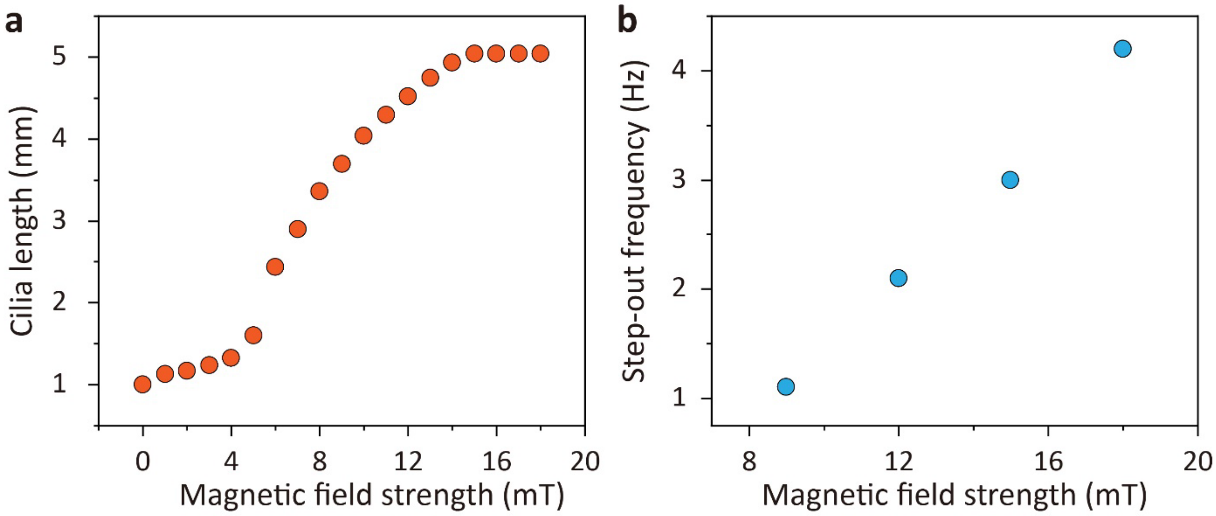

Figure S27. The performance of liquid cilia. a. The relationship between liquid cilia length and magnetic field strength. b. Cut-off frequency of liquid cilia array (1×7) versus magnetic field strength (rotation angle of 120°). The length of artificial cilia based on ferrofluid droplets can be adjusted (determined by the strength of the external magnetic field) compared to artificial cilia based on magnetic elastomers. We know that the farther away from the cilia position, the smaller the fluid velocity. While the solid cilia's length is fixed, it isn't easy to reconfigure it to meet the fluid pumping farther away from the cilia. The adjustable length of the liquid cilia allows the liquid cilia to be reconfigured in situ for more flexible fluid pumping. As shown in Figure S27a, the size of the static liquid cilia can be elongated to 5 times the initial length (1 mm) under the magnetic field of 18 mT. In addition, the step-out frequency of liquid cilia based on ferrofluid droplets is determined by the magnetic strength. The cut-off frequency is 1.1 Hz when the magnetic field strength is 9 mT; when the magnetic field strength increases to 18 mT, the cut-off frequency is about 4.2 Hz (Figure S27b). And the pumping speed of liquid cilia is not determined by the frequency alone. We can also increase the pumping speed by adjusting the external magnetic field to increase the cilia length and oscillation angle. For example, in the previous response letter, when the magnetic field strength is 9 mT, the cut-off frequency of the cilia array is 1.1 Hz, the length of dynamic cilia is 1.2 mm, and when its oscillation angle is set as 120°, its pumping fluid velocity is about 0.195 mm/s. However, when the magnetic field strength is increased to 18 mT, the cut-off frequency of the cilia array is 4.2 Hz, the length of dynamic cilia is about 1.5 mm, and when the oscillation angle is increased to 150°, and finally, the pumping velocity will increase to 0.97 mm/s.

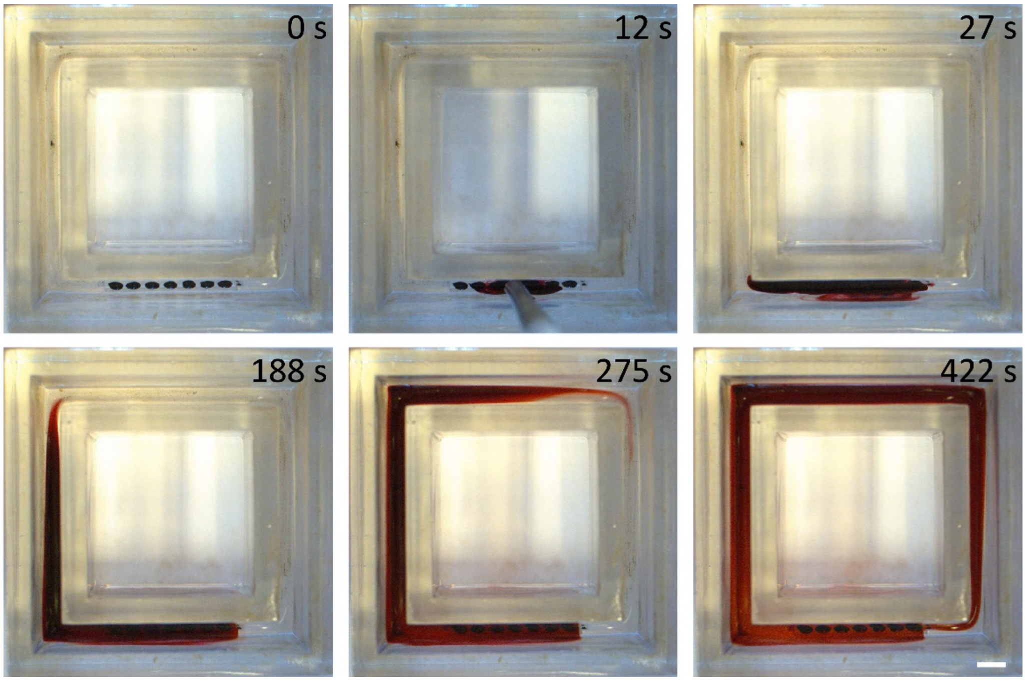

Figure S28. Functional liquid cilia as fluidic devices. As a concept demonstration, a square closed channel is designed with a length of 45 mm, a width of 5 mm, and a depth of 15 mm. A one-dimensional array of seven liquid cilia is placed at the center of one side of the square closed channel. It oscillates in a right-to-left direction (*t* = 0 s) under the guidance of a magnetic field BCilia(t) (*f* = 1 Hz and Bm = 9 mT). The edible stain is added directly above the oscillating liquid cilia (*t* = 12 s). After 410 s of oscillation, the edible dye is pumped once along the channel from the starting point to the starting position, forming a closed-loop (*t* = 422 s). The speed of pumping liquid is about 0.43 mm/s. Scale bar, 5 mm.

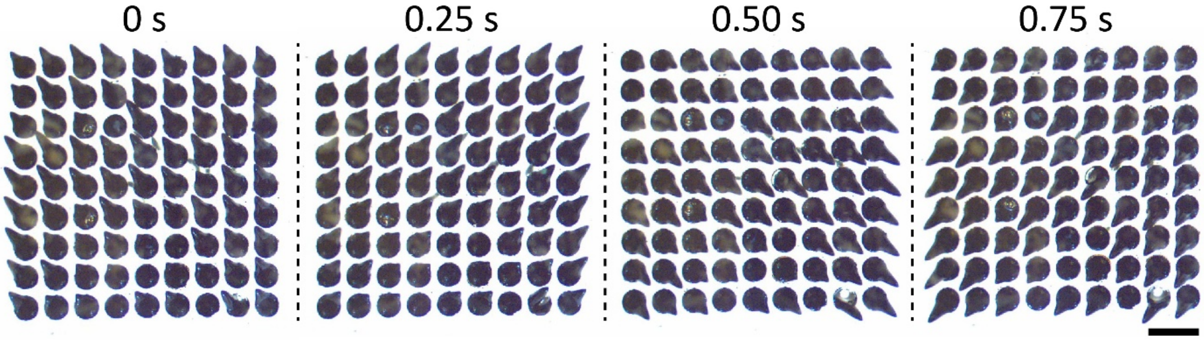

Figure S29. Top views of liquid cilia matrix with 2D synchronous waves. By regulating the direction of rotation of the external magnetic field, the direction of rotation of the liquid cilia matrix can be controlled. By applying a three-dimensional conical magnetic field, all the liquid cilia on the liquid cilia matrix rotate around the bottom in a circular motion. The angle of the liquid cilia changes from 135°to -135°from 0 s to 0.75 s. In addition, the angle between the liquid cilia and the base plane is also controlled by the external magnetic field. This synchronous rotation of the liquid cilia induces the generation of a fluid field that accelerates the mixing of the fluid. Scale bar, 5 mm.

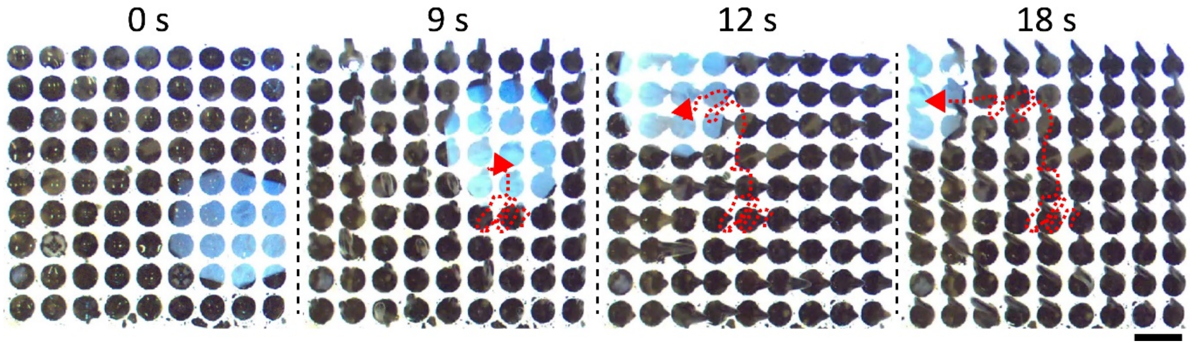

Figure S30. Snapshots show an irregular object's directional motion by the liquid cilia matrix. Liquid cilia matrix enables amphibious cargo transport, in addition to pumping liquids such as water or blood, but also solids. Due to its elongation-rotation-contraction motion mechanism, liquid cilia do not have a recovery stroke. Therefore, the liquid cilia matrix can exert a directional force on the solid particles above it and does not cause it to have a reciprocal motion. When $t = 0$ s, an irregular object with a volume of about 10 mm×10 mm×2 mm is located in the lower right corner of the liquid cilia matrix. Then the liquid cilia matrix is controlled to swing upward and to the left side, respectively. After 18 s, the irregular object is finally transported to the top left by the liquid cilia matrix. The red dashed line indicates the trajectory of the irregular object. Scale bar, 5 mm.

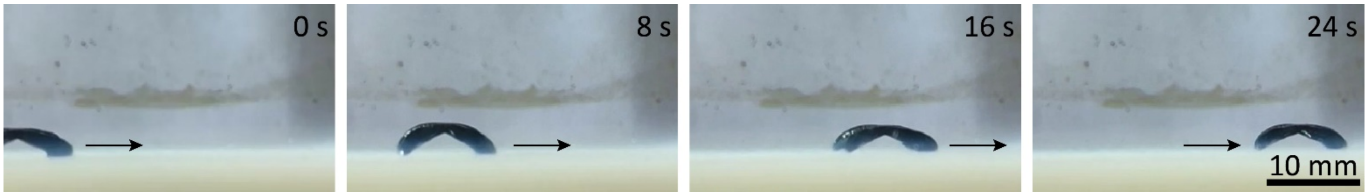

Figure S31. Snapshot showing a four-legged spider robot walking directionally. A ferrofluid droplet with a diameter of 10 mm is controllably split into multiple sub-droplets. An adhesion strategy transforms a four-flap silicone elastomer sheet into a spider robot. The spider robot exhibits directional walking under a low-frequency rotating magnetic field. At $t = 0$ s, the spider robot is located on the leftmost side. After about 24 s, the spider robot reaches the rightmost side and walks a distance of about 30 mm with an average speed of about 1.25 mm/s. The black arrow indicates the direction of motion. Scale bar, 10 mm.

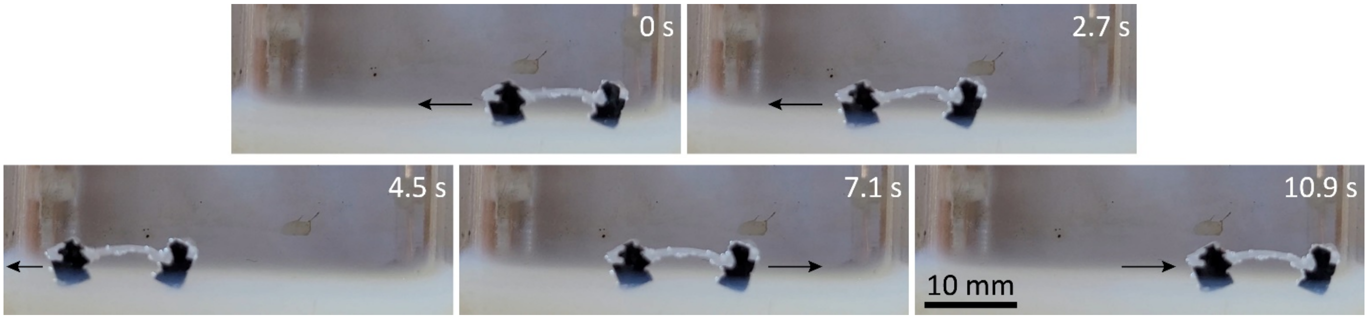

Figure S32. Side view of the crawling caterpillarbot. A ferrofluid droplet with a diameter of 5 mm is controllably split into two sub-droplets and transforms a silicone elastomer sheet into a caterpillar robot through an adhesion strategy. Two sub-droplets are controllably adhered to the ends of the silicone elastomer, forming the caterpillar robot. The caterpillar robot exhibits directional crawling motion mode under an oscillating magnetic field. At t = 0 s, the caterpillar robot is located at the rightmost side. After about 4.5 s, the caterpillar robot reaches the leftmost side and crawls a distance of about 30 mm with an average speed of about 6.67 mm/s. By subsequently changing the oscillating direction of the oscillating magnetic field, the caterpillar robot can crawl in reverse without shifting its body. After 6.4 s, it reaches the rightmost side from the leftmost side. Scale bar, 10 mm.

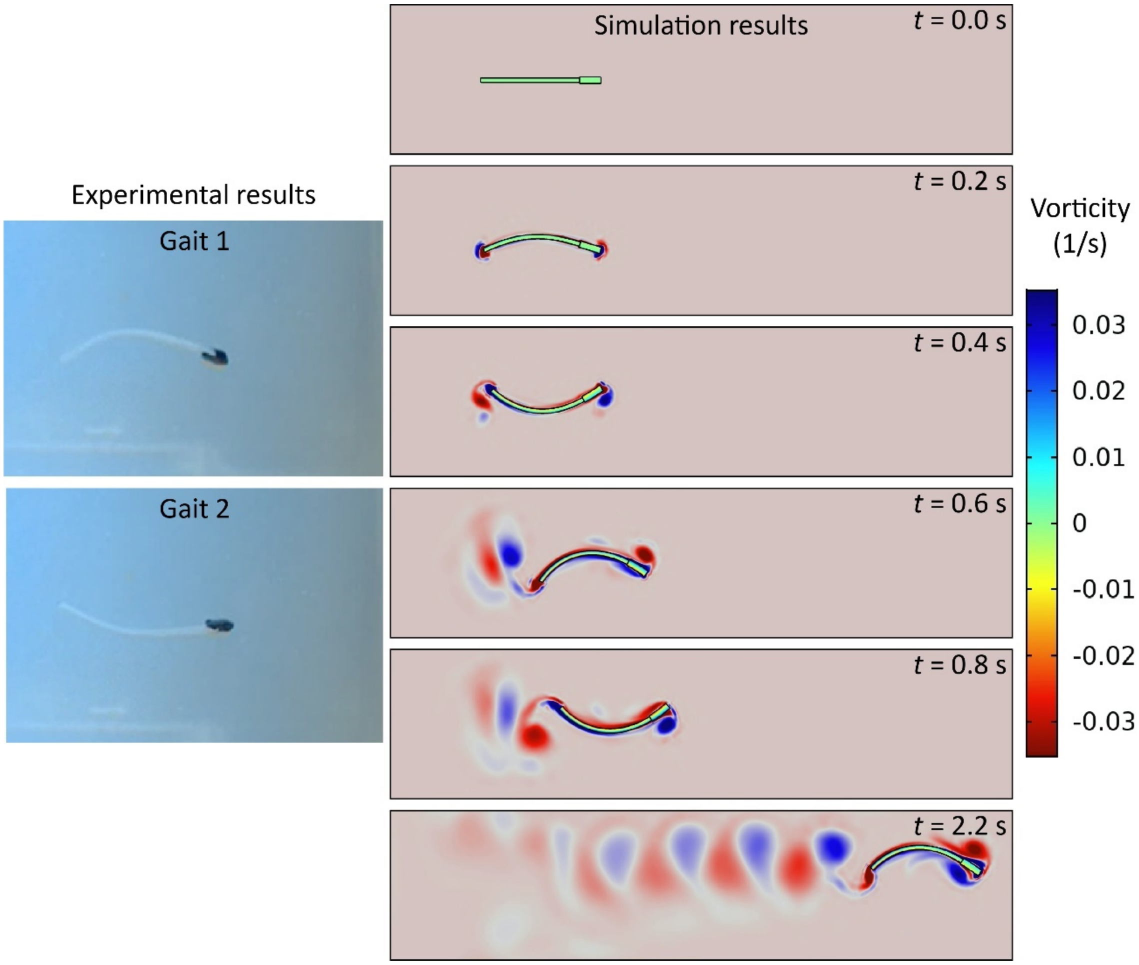

Figure S33. The experimental results and simulation results of the gait of the 'fish robot'. The head of the fish robot is firstly wetted by the ferrofluid droplet, which enables it to be controlled by the external magnetic field. The oscillating magnetic field will force the robot's head to sway from side to side, a process similar to excitation at one terminal of a beam at its first-order bending vibration frequency, thus causing the beam to bend. Since the head is heavier and more rigid (since the elastomer sheet at this region is wetted by the ferrofluid droplet), the tail displacement will be more pronounced. Then when the tail swings from side to side, the water on the rear side is continuously pushed away from the body, causing vortices behind its tail as the COMSOL Multiphysics simulation result, and the reaction force generated by the fluid on the robot's body during this process will eventually push the robot forward. The Fluid-Structure Interaction module is applied, the length of the magnetic head of the fish robot is set to 5 mm and the thickness is 1.5 mm, and the length of the non-magnetic tail is set to 23 mm and the thickness is 1 mm. The oscillation frequency of the head of the fish robot is 4 Hz. The relationship between the deformation of the fish robot body and the fluid force is given by $\rho_s \ddot{\mathbf{u}}_s = div\ \mathbf{S} + \mathbf{f}_v$, where ρ_s is the mass density of the fish robot, $\mathbf{u}_s$ is the displacement vector, $\mathbf{S}$ is the reference stress applied to the fish robot, $\mathbf{f}_v$ is the fluid force exerted on the solid structure. The fluid is governed by the forces balance and mass conservation equations as follows: $\rho_f \dot{\boldsymbol{v}}_f + \rho_f(\nabla \mathbf{v}_f)\mathbf{v}_f = div\ \Gamma + \mathbf{f}$ and $\dot{\rho}_f + \mathrm{div}\left(\rho_f \mathbf{v}_f\right) = 0$, Where ρ_f is the mass density of the fluid, $\mathbf{v}_f$ is the fluid spatial velocity, $\mathbf{f}$ is the force of solid acting on the fluid, and the stress Γ is given by:

$\Gamma = -pI + 2\mu_f(sym\ \nabla \mathbf{v}_f) - \frac{2}{3}\mu_f(div\ \mathbf{v}_f)\mathbf{I}$, where p is the fluid pressure, and μ_f is the dynamic viscosity.

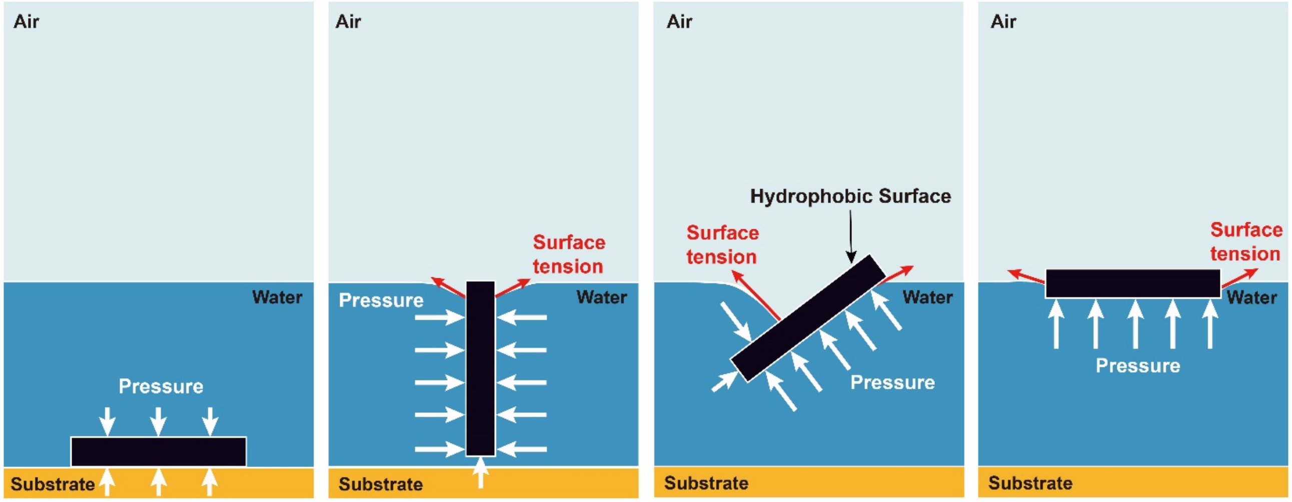

Figure S34. The mechanism by which the robot rises to the surface. In the initial state, the robot will sink at the bottom of the tank because it is denser than water. When the magnetic field direction becomes vertical, the robot will align to this direction, and its head will poke out of the water. Due to its hydrophobicity, the out-of-water part will not suffer the hydrostatic pressure, and the surface tension will further pull the robot towards the water-air interface. Eventually, the robot lies flat on the water surface due to the surface tension and buoyancy.

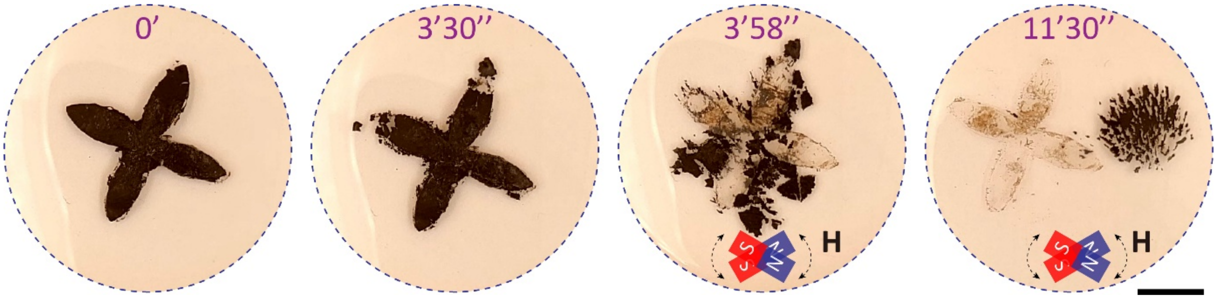

Figure S35. Photo images depict the shedding and disintegration process of liquid exoskeleton from the robot. Scale bars, 10 mm.

References	Normalized velocity<br>v/fL	Fluid velocity<br>v	Driving frequency<br>f	Cilia length<br>L	Cilia number<br>N	Materials
1	0.1	3.3 µm/s	1 Hz	31 µm	3×3	Superparamagnetic microparticles
2	0.143	500 µm/s	7 Hz	500 µm	1×6	Polydimethylsiloxane
3	0.0214	75 µm/s	10 Hz	350 µm	3×3	Polydimethylsiloxane
4	0.011	9 µm/s	34 Hz	25 µm	3000	Silicone elastomer
5	0.25	83 µm/s	0.083 Hz	4 mm	8×8	Silicone elastomer
6	0.38	0.95 mm/s	2.5 Hz	1 mm	6×6	Silicone elastomer
**This work**	**0.154**	**0.97 mm/s**	**4.2 Hz**	**1.5 mm**	**1×7**	**Ferrofluid**

Table S1. Comparison of the fluid pumping performance using artificial cilia with literature. The normalized velocity, shows how fast the flow it can generate by one beating cycle. The magnetic field strength used for this work is 18 mT and the rotation angle of the liquid cilia is 150°.

Supplementary References

1. Vilfan, M., Potočnik, A., Kavčič, B., Osterman, N., Poberaj, I., Vilfan, A., Babič, D. Self-assembled artificial cilia. *Proc. Natl. Acad. Sci. U. S. A.* **2010**, *107*, 1844-1847.

2. Rockenbach, A., Schnakenberg, U. The influence of flap inclination angle on fluid transport at ciliated walls. *J. Micromechanics Microengineering* **2017**, *27*, 015007.

3. Zhang, S., Wang, Y., Lavrijsen, R., Onck, P. R., den Toonder, J. M. Versatile microfluidic flow generated by moulded magnetic artificial cilia. *Sensors Actuators B Chem.* **2018**, *263*, 614-624.

4. Shields, A. R., Fiser, B. L., Evans, B. A., Falvo, M. R., Washburn, S., Superfine, R. Biomimetic cilia arrays generate simultaneous pumping and mixing regimes. *Proc. Natl. Acad. Sci. U. S. A.* **2010**, *107*, 15670-15675.

5. Gu, H., Boehler, Q., Cui, H., Secchi, E., Savorana, G., De Marco, C., Gervasoni, S., Peyron, Q., Huang, T., Pane, S., Hirt, A. M., Ahmed, D., Nelson, B. J. Magnetic cilia carpets with programmable metachronal waves. *Nat. Commun.* **2020**, *11*, 1-10.

6. Dong, X., Lum, G. Z., Hu, W., Zhang, R., Ren, Z., Onck, P. R., Sitti, M. Bioinspired cilia arrays with programmable nonreciprocal motion and metachronal coordination. *Sci. Adv.* **2020**, *6*, eabc9323.

Supplementary Movies

Movie S1. Torque-driven multimodal motion of ferrofluid droplets.

Movie S2. Fission and fusion of ferrofluid droplets.

Movie S3. Multimodal locomotion over artificial unstructured environments.

Movie S4. Multimodal locomotion over biological environments.

Movie S5. Controllable liquid capsule for cargo transportation and release.

Movie S6. Navigation of capsules under real-time medical imaging.

Movie S7. Ferrofluid droplets as programmable liquid cilia.

Movie S8. Ferrofluid droplets as liquid leg.

Movie S9. Ferrofluid droplets as smart skin.

Movie S10. Ferrofluid droplet traverses complex maze to transform elastomeric sheet into robot.
