# Supplementary material for: Exploiting ferrofluidic wetting for miniature soft machines
Source: Nat Commun. 2022 Dec 23;13:7919. doi: 10.1038/s41467-022-35646-y (PMC9789085; doi:10.1038/s41467-022-35646-y)
Supplement: Supplementary file 3 — Description of Additional Supplementary Files [file 41467_2022_35646_MOESM3_ESM.pdf]

Description of Additional Supplementary Files

File Name: Supplementary Movie 1
Description: Torque-driven multimodal motion of ferrofluid droplets.

File Name: Supplementary Movie 2
Description: Fission and fusion of ferrofluid droplets.

File Name: Supplementary Movie 3
Description: Multimodal locomotion over artificial unstructured environments.

File Name: Supplementary Movie 4
Description: Multimodal locomotion over biological environments.

File Name: Supplementary Movie 5
Description: Controllable liquid capsule for cargo transportation and release.

File Name: Supplementary Movie 6
Description: Navigation of capsules under real-time medical imaging.

File Name: Supplementary Movie 7
Description: Ferrofluid droplets as programmable liquid cilia.

File Name: Supplementary Movie 8
Description: Ferrofluid droplets as liquid legs.

File Name: Supplementary Movie 9
Description: Ferrofluid droplets as smart skin.

File Name: Supplementary Movie 10
Description: Ferrofluid droplet traverses complex maze to transform elastomeric sheet into robot.
