# Supplementary material for: Exploiting ferrofluidic wetting for miniature soft machines
Source: Nat Commun. 2022 Dec 23;13:7919. doi: 10.1038/s41467-022-35646-y (PMC9789085; doi:10.1038/s41467-022-35646-y)
Supplement: Supplementary file 14 — Reporting Summary [file 41467_2022_35646_MOESM14_ESM.pdf]

nature portfolio

Corresponding author(s): Carmel Majidi, Li Zhang

Last updated by author(s): Dec 15, 2022

Reporting Summary

Nature Portfolio wishes to improve the reproducibility of the work that we publish. This form provides structure for consistency and transparency in reporting. For further information on Nature Portfolio policies, see our Editorial Policies and the Editorial Policy Checklist.

Statistics

For all statistical analyses, confirm that the following items are present in the figure legend, table legend, main text, or Methods section.

n/a	Confirmed	
☐	☒	The exact sample size (*n*) for each experimental group/condition, given as a discrete number and unit of measurement
☐	☒	A statement on whether measurements were taken from distinct samples or whether the same sample was measured repeatedly
☒	☐	The statistical test(s) used AND whether they are one- or two-sided<br>*Only common tests should be described solely by name; describe more complex techniques in the Methods section.*
☒	☐	A description of all covariates tested
☒	☐	A description of any assumptions or corrections, such as tests of normality and adjustment for multiple comparisons
☐	☒	A full description of the statistical parameters including central tendency (e.g. means) or other basic estimates (e.g. regression coefficient) AND variation (e.g. standard deviation) or associated estimates of uncertainty (e.g. confidence intervals)
☒	☐	For null hypothesis testing, the test statistic (e.g. *F*, *t*, *r*) with confidence intervals, effect sizes, degrees of freedom and *P* value noted<br>*Give P values as exact values whenever suitable.*
☒	☐	For Bayesian analysis, information on the choice of priors and Markov chain Monte Carlo settings
☒	☐	For hierarchical and complex designs, identification of the appropriate level for tests and full reporting of outcomes
☒	☐	Estimates of effect sizes (e.g. Cohen's *d*, Pearson's *r*), indicating how they were calculated

Our web collection on statistics for biologists contains articles on many of the points above.

Software and code

Policy information about availability of computer code

Data collection	The fluid simulation of liquid cilia was performed using COMSOL Multiphysics 5.5<br>Medical ultrasound imaging was collected using Terason t3200<br>X-ray imaging was collected from Artis zeego
Data analysis	Matlab 2017a was used for the data analysis<br>Image J 1.5

For manuscripts utilizing custom algorithms or software that are central to the research but not yet described in published literature, software must be made available to editors and reviewers. We strongly encourage code deposition in a community repository (e.g. GitHub). See the Nature Portfolio guidelines for submitting code & software for further information.

Data

Policy information about availability of data

All manuscripts must include a data availability statement. This statement should provide the following information, where applicable:

- Accession codes, unique identifiers, or web links for publicly available datasets
- A description of any restrictions on data availability
- For clinical datasets or third party data, please ensure that the statement adheres to our policy

The authors declare that data supporting the findings of this study are available within the paper and its Supplementary Information. Source data are provided with this paper.

Human research participants

Policy information about studies involving human research participants and Sex and Gender in Research.

Reporting on sex and gender	Sex and gender information was not considered and collected because the experiments were performed using only the complex lumen structure of the neonatal placenta, which is not correlated with sex and gender.
Population characteristics	Not relevant.
Recruitment	All enrolled patients provided written informed consent. The human placenta was donated by pregnant women in collaboration with the Department of Obstetrics and Gynaecology (CUHK).
Ethics oversight	The placentas of the newborn babies used in the experiments were collected from the Prince of Wales Hospital, Hong Kong. The collection of human placentas was approved and overseen by The Joint Chinese University of Hong Kong-New Territories East Cluster Clinical Research Ethics Committee (The Joint CUHK-NTEC CREC) (Ref. No. 2020.384).

Note that full information on the approval of the study protocol must also be provided in the manuscript.

Field-specific reporting

Please select the one below that is the best fit for your research. If you are not sure, read the appropriate sections before making your selection.

☒ Life sciences ☐ Behavioural & social sciences ☐ Ecological, evolutionary & environmental sciences

For a reference copy of the document with all sections, see nature.com/documents/nr-reporting-summary-flat.pdf

Life sciences study design

All studies must disclose on these points even when the disclosure is negative.

Sample size	The presented results are based on secondary experiments, while the first experiments were used to observe the sample variations and define sample size.
Data exclusions	No data acquired for quantitative analysis were excluded.
Replication	All the experimental findings were replicated with the corresponding sample sizes as indicated in the caption of the relevant figure. All these findings were replicated successfully at different times.
Randomization	The imaging of all the samples was randomly allocated.
Blinding	A blind approach in this experiment was not possible since the experiments were visible. However, all the samples were collected and analyzed under the same condition.

Reporting for specific materials, systems and methods

We require information from authors about some types of materials, experimental systems and methods used in many studies. Here, indicate whether each material, system or method listed is relevant to your study. If you are not sure if a list item applies to your research, read the appropriate section before selecting a response.

Materials & experimental systems

n/a	Involved in the study
☒	☐ Antibodies
☐	☒ Eukaryotic cell lines
☒	☐ Palaeontology and archaeology
☒	☐ Animals and other organisms
☒	☐ Clinical data
☒	☐ Dual use research of concern

Methods

n/a	Involved in the study
☒	☐ ChIP-seq
☒	☐ Flow cytometry
☒	☐ MRI-based neuroimaging

Eukaryotic cell lines

Policy information about cell lines and Sex and Gender in Research

Cell line source(s)	NIH/3T3 is a fibroblast cell line that was isolated from a mouse NIH/Swiss embryo. The cell line NIH/3T3 was used for the cell viability studies.
Authentication	None of the cell lines used were authenticated.
Mycoplasma contamination	It was confirmed as negative for Mycoplasma contamination.
Commonly misidentified lines (See ICLAC register)	According to ICLAC this is not a commonly misidentified cell line.
